# Antiproliferative and Cytotoxic Activities of Fluorescein—A Diagnostic Angiography Dye

**DOI:** 10.3390/ijms23031504

**Published:** 2022-01-28

**Authors:** Mária Šranková, Aleš Dvořák, Marek Martínek, Peter Šebej, Petr Klán, Libor Vítek, Lucie Muchová

**Affiliations:** 1 Institute of Medical Biochemistry and Laboratory Diagnostics, General University Hospital in Prague and 1st Faculty of Medicine, Charles University, Na Bojišti 3, 121 08 Praha, Czech Republic; majka.srankova@gmail.com (M.Š.); aleshdvorak@gmail.com (A.D.); 2 RECETOX, Faculty of Science, Masaryk University, Kotlářská 2, 611 37 Brno, Czech Republic; marek.martinek@gmail.com (M.M.); sebej@recetox.muni.cz (P.Š.); klan@sci.muni.cz (P.K.); 3 Department of Chemistry, Faculty of Science, Masaryk University, Kamenice 5, 625 00 Brno, Czech Republic; 4 4th Department of Internal Medicine, General University Hospital in Prague and 1st Faculty of Medicine, Charles University, U Nemocnice 2, 128 08 Praha, Czech Republic

**Keywords:** fluorescein, irradiation, singlet oxygen, carbon monoxide, viability, metabolism, proliferation

## Abstract

Fluorescein is a fluorescent dye used as a diagnostic tool in various fields of medicine. Although fluorescein itself possesses low toxicity, after photoactivation, it releases potentially toxic molecules, such as singlet oxygen (^1^O_2_) and, as we demonstrate in this work, also carbon monoxide (CO). As both of these molecules can affect physiological processes, the main aim of this study was to explore the potential biological impacts of fluorescein photochemistry. In our in vitro study in a human hepatoblastoma HepG2 cell line, we explored the possible effects on cell viability, cellular energy metabolism, and the cell cycle. We observed markedly lowered cell viability (≈30%, 75–2400 μM) upon irradiation of intracellular fluorescein and proved that this decrease in viability was dependent on the cellular oxygen concentration. We also detected a significantly decreased concentration of Krebs cycle metabolites (lactate and citrate < 30%; 2-hydroxyglutarate and 2-oxoglutarate < 10%) as well as cell cycle arrest (decrease in the G2 phase of 18%). These observations suggest that this photochemical reaction could have important biological consequences and may account for some adverse reactions observed in fluorescein-treated patients. Additionally, the biological activities of both ^1^O_2_ and CO might have considerable therapeutic potential, particularly in the treatment of cancer.

## 1. Introduction

Fluorescein is a fluorescent small-molecule organic dye that is commonly employed in cellular biology as a tracer. Its sodium salt is widely used in clinical medicine, particularly as a diagnostic tool in ophthalmology [1]. It has also been studied for new therapeutic applications in various fields, such as urology [2,3,4] and neurosurgery [5,6]. Having an important role in the diagnostics of ocular diseases, fluorescein has been included on the List of Essential Medicines, published by the World Health Organization [7]. During diagnostic procedures, it can be administered locally [8], orally [9], or intravenously [10] with subsequent irradiation of the area of interest using blue light [1,3,4] (≈490 nm [11,12]).

Although fluorescein is considered to be generally safe, it is a photoactive compound whose biological effects associated with this activity have been neglected to date. For example, fluorescein is known to photosensitize oxygen to form singlet oxygen (^1^O_2_) [13,14]. Furthermore, some of us have demonstrated that a fluorescein analog, xanthene-9-carboxylic acid, releases carbon monoxide (CO) upon photoactivation by green light [15] via decarbonylation of the carboxyl group. Although fluorescein is structurally different, we hypothesized that it could also undergo the photodecarbonylation reaction.

Both ^1^O_2_ and CO are biologically active molecules that affect physiological processes in the human body [16,17,18]. Over the past three decades, they have also been thoroughly studied for their use in the treatment of various diseases. ^1^O_2_ is a very reactive molecule, the cytotoxic properties of which are utilized in medicine, e.g., in photodynamic therapy [19]. CO is an important gasotransmitter with anti-inflammatory [20], antiapoptotic [21], and antiproliferative properties [22]. Its anticancer action was also studied in our laboratory, showing a positive effect on the survival rate of mice xenotransplanted with pancreatic cancer [23]. Although ^1^O_2_ and CO are being investigated for their potential therapeutic applications, both exert cytotoxicity at higher concentrations, particularly when their transport to target sites is not strictly controlled. While ^1^O_2_ causes oxidative damage and cell death [24,25,26,27], the toxicity of CO is related to its high binding affinity to blood hemoglobin [28,29,30,31] or the heme moiety of extravascular hemoproteins [32,33] such as cytochrome c oxidase [34], affecting their oxygen carrier properties or enzymatic activities, respectively. In addition, CO can trigger oxidative stress [35] and lipoperoxidation [36].

In this paper, we focus on the findings that irradiation of fluorescein in aqueous solutions produces not only ^1^O_2_ but also CO, as also demonstrated in our parallel article describing the photochemical behavior of common xanthene dyes [37]. Due to the increasing mass of evidence indicating the possible biological activities of both ^1^O_2_ and CO, our goal was to evaluate the biological consequences of fluorescein photochemistry using an in vitro hepatic cell model, specifically its toxicity, as well as its effects on both cellular metabolism and the cell cycle. We attempted to provide improved insight into the impact of fluorescein irradiation on biological systems, to draw attention to its potentially harmful effects in the human body, and to point out the therapeutic potential of fluorescein when the photochemical reaction is controlled.

## 2. Results

### 2.1. CO and ^1^O_2_ Release upon Fluorescein Photoexcitation

To test the hypothesis that both CO and ^1^O_2_ are released upon fluorescein photoexcitation, fluorescein solution was irradiated with white light, and the amounts of both gaseous molecules produced were quantified. We proved that under these experimental conditions, ^1^O_2_ was released with a quantum yield of φ = 0.018 (median, IQR = 0.012; Figure S1, Table S1).

Moreover, we demonstrated that the photoreaction of fluorescein led to the production of CO in a chemical yield of 40% (GC-RGA and GC-MS; Table S2).

### 2.2. CO Content in HepG2 Cells

As fluorescein shows a very low penetrability across the cell membrane, fluorescein diacetate (FDA), a more lipophilic fluorescein derivative capable of penetrating cell membranes, was used to distinguish between the intra- and extracellular effects of fluorescein photochemical reactions. Intracellularly, FDA is immediately converted to fluorescein by cytoplasmic esterases [38]. As FDA is a colorless, non-fluorescent dye, its intracellular conversion to fluorescein can be determined directly by fluorescent microscopy (Figure S2).

**Figure 1:**
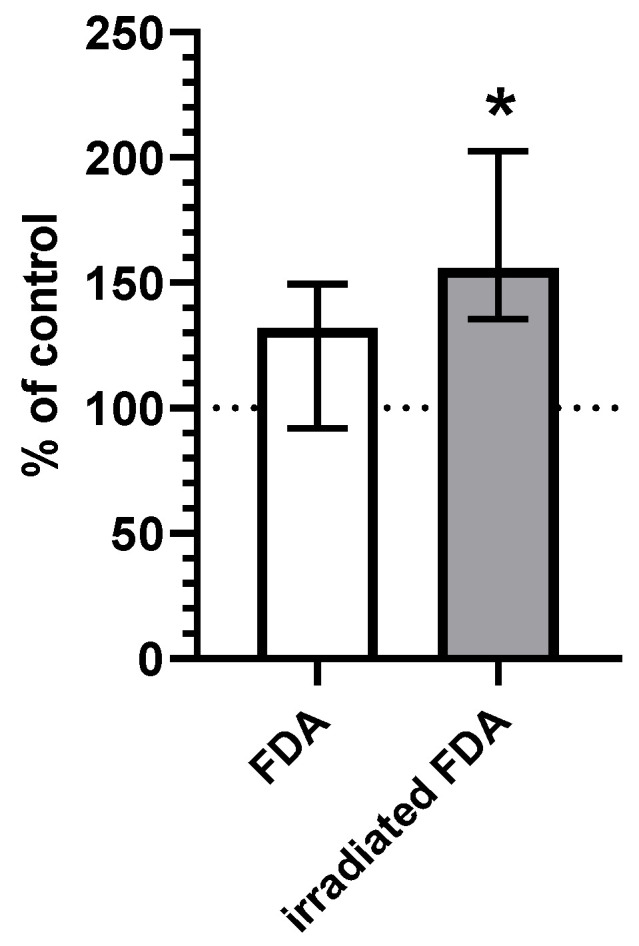
CO concentration in HepG2 cells incubated for 2 h with an irradiated FDA solution (c(FDA) = 600 μmol/L, irradiation throughout the entire incubation time, I = 600 mW/cm^2^, n = 6) or a non-irradiated FDA solution (c(FDA) = 600 μmol/L, incubated in the dark, n = 6). Data are expressed as % of a control (100%); * *p* ≤ 0.05 vs. control.

To demonstrate that CO can be released from fluorescein intracellularly, the CO production was measured in HepG2 cells after treatment with FDA. Irradiated cells treated with FDA showed a significant increase in the CO content compared to the untreated/non-irradiated controls (Figure 1).

### 2.3. Cell Viability

#### 2.3.1. Viability of HepG2 Cells Treated with FDA or Fluorescein Photoproducts

**Figure 2:**
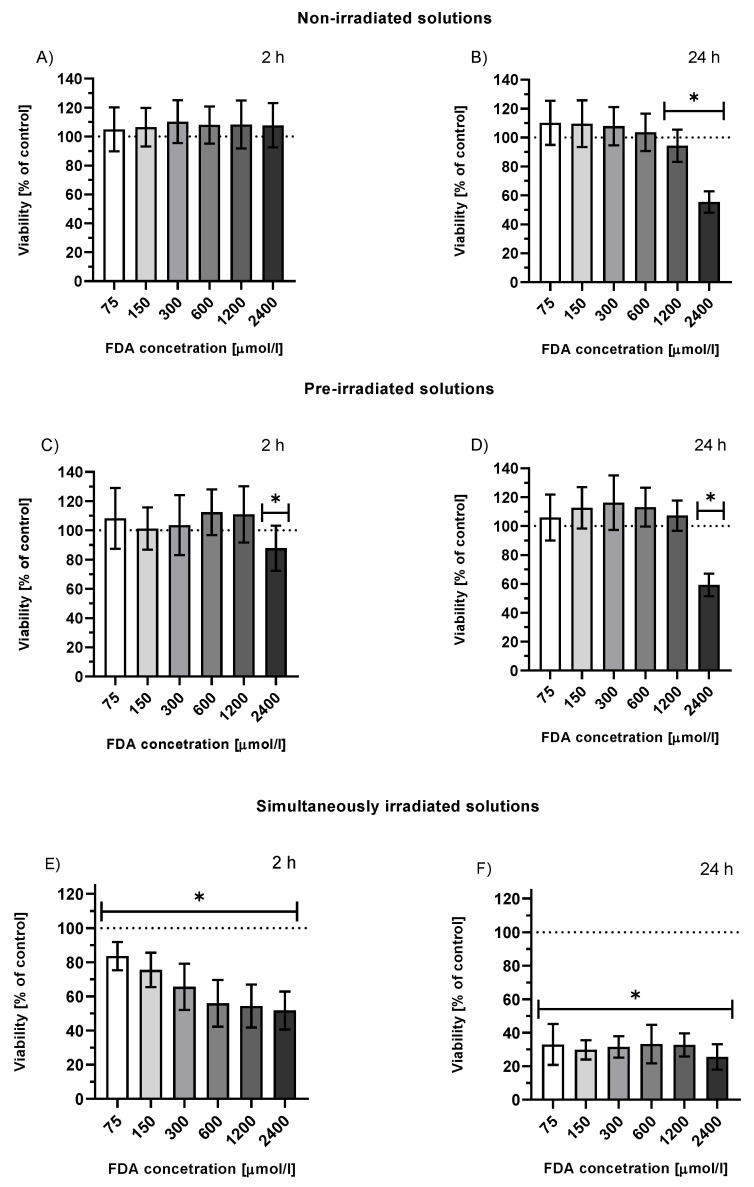
Viability of HepG2 cells treated with solutions of non-irradiated FDA solution (**A**,**B**), solution of fluorescein photoproducts (**C**,**D**) (t_ir_ = 24 h, I = 160 mW/cm^2^ prior to the treatment, FDA concentration = initial concentration of FDA prior to irradiation), or simultaneously irradiated solutions of FDA (**E**,**F**) (irradiation throughout the entire incubation time 2 or 24 h, I = 160 mW/cm^2^, FDA concentration = FDA initial concentration prior to irradiation); n ≥ 20; * *p* ≤ 0.05 vs. untreated control.

The treatment of HepG2 cells with FDA showed no cytotoxicity after 2 h within the whole tested concentration range or up to an FDA concentration of 600 µmol/L if treated for 24 h. Stable fluorescein photoproducts obtained upon exhaustive irradiation of an FDA solution did not affect cell viability up to a concentration of 1.2 mmol/L after 2 h and 24 h incubation (Figure 2A–D).

#### 2.3.2. Viability of Irradiated HepG2 Cells Treated with FDA or Fluorescein

In the subsequent set of experiments, we investigated the effects of irradiation on cells treated with FDA (with high levels of membrane penetration; i.e., when fluorescein is released upon hydrolysis in cells, localized intracellularly) or fluorescein sodium salt (low membrane penetration, mainly extracellular localization). Irradiation of FDA-treated HepG2 cells for 2 or 24 h resulted in a dramatic decrease in cell viability within a concentration range of 75–2400 μmol/L (Figure 2E,F).

**Figure 3:**
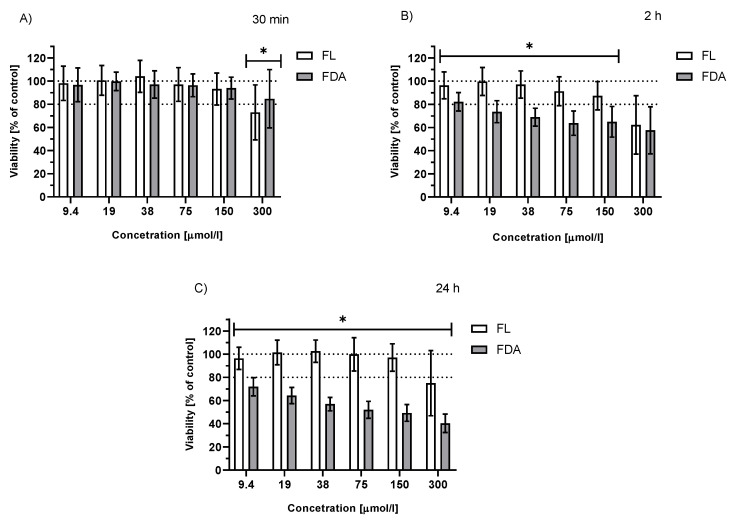
Comparison of the effect of FDA and fluorescein sodium salt on the viability of HepG2 cells. Treated cells (c_sol_ = 9.4–300 µmol/L) were irradiated with white light (LED, I = 160 mW/cm^2^) for 30 min and then kept in the dark. Analyzed: (**A**) immediately after irradiation; (**B**) after 2 h; (**C**) after 24 h; n ≥ 24; * *p* ≤ 0.05 (fluorescein vs. FDA).

To obtain more clinically relevant data, as diagnostic fluorescein angiography lasts only a few minutes, HepG2 cells treated with FDA or fluorescein were irradiated for 30 min and analyzed immediately or after further incubation for 2 or 24 h in the dark (Figure 3). Irradiation of cells treated with FDA showed a significant decrease in cell viability (≤80%) at the majority of the analyzed concentrations (19–300 μmol/L) for 2 and 24 h incubation intervals, whereas this effect was much weaker when cells were treated with fluorescein (Figure 3).

#### 2.3.3. Viability of Irradiated HepG2 Cells Treated with FDA and Fluorescein under Hypoxemic Conditions

**Figure 4:**
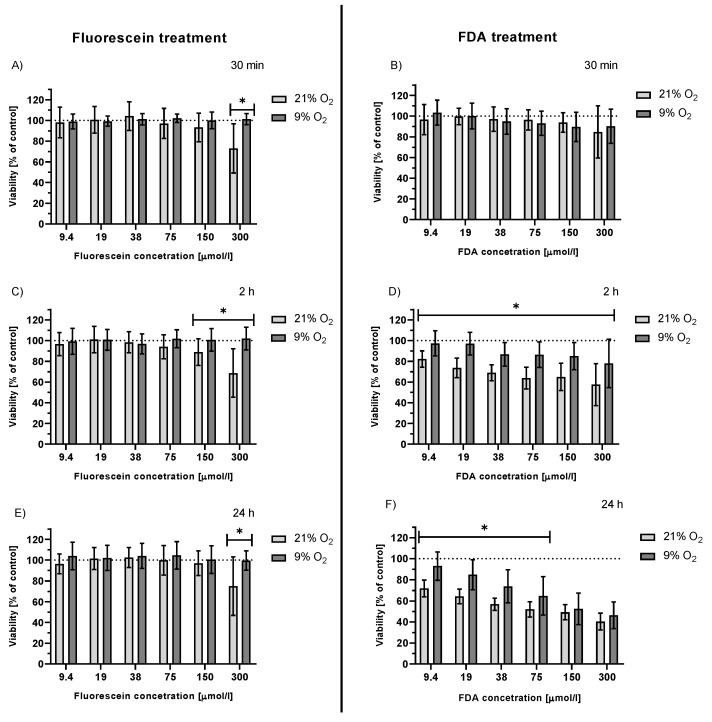
Viability of HepG2 cells treated with fluorescein (**A**,**C**,**E**) or FDA (**B**,**D**,**F**) kept under two different atmospheric oxygen concentrations (21 and 9% O_2_). Treated cells were irradiated with white light (LED, I = 160 mW/cm^2^) for 30 min and kept in the dark. Analyzed: (**A**,**B**) immediately after irradiation; (**C**,**D**) after 2 h; and (**E**,**F**) after 24 h; n ≥ 24; * *p* ≤ 0.05 (normoxia vs. hypoxia).

To analyze the effect of the oxygen concentration on the cytotoxicity of fluorescein and FDA, HepG2 cells were treated and irradiated in a hypoxic chamber (9% O_2_ level). During hypoxia, less ^1^O_2_ was produced upon fluorescein irradiation (Figure S3); thus, this parameter might have a significant effect on ^1^O_2_-induced cytotoxicity. In this experimental setup, significantly lower cytotoxicity of FDA was observed compared to that under normoxic conditions (Figure 4B,D,F). In the case of fluorescein, the cytotoxicity observed in 21% atmospheric oxygen diminished with decreasing O_2_ concentration (Figure 4A,C,E).

### 2.4. Effect of Fluorescein Photoreaction on Krebs Cycle Metabolites

#### 2.4.1. FDA Solution

**Figure 5:**
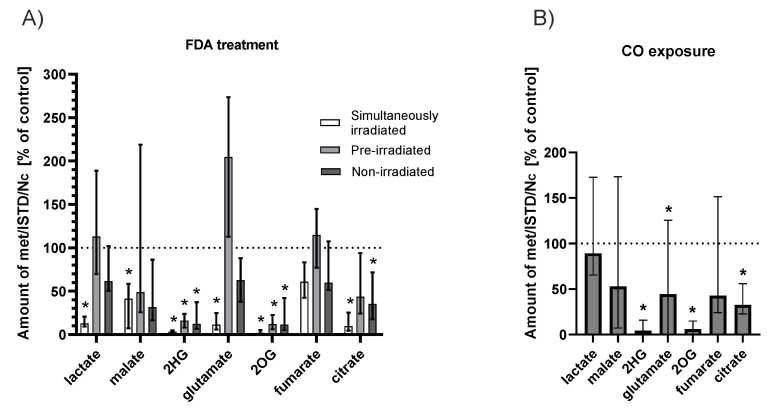
The effect of fluorescein irradiation (**A**) and CO atmosphere (**B**, 100 ppm) on Krebs cycle intermediates levels in HepG2 cells. (**A**) HepG2 cells treated for 2 h with non-irradiated (c(FDA) = 150 μmol/L), pre-irradiated (pre-irradiation for 24 h prior to the treatment, c(FDA) = 150 μmol/L, I = 160 mW/cm^2^), and simultaneously irradiated solutions of FDA (irradiation during the entire incubation time, I = 160 mW/cm^2^, c(FDA) = 150 μmol/L); (**B**) cells exposed to an atmosphere enriched with gaseous CO (100 ppm) for 2 h. The values determined by an internal-standard method (ISTD) are relative to the cell number (N_C_) and are expressed as a % of untreated controls; (**A**) n = 9, (**B**) n = 12; * *p* ≤ 0.05 vs. controls (control = untreated cells); 2HG, 2-hydroxyglutarate; 2OG, 2-oxoglutarate.

To elucidate the effects of fluorescein irradiation on cellular metabolism, the concentrations of Krebs cycle intermediates and its anaplerotic pathways were analyzed in HepG2 cells. We examined the effects of fluorescein itself as well as its photoproducts, which were prepared by exhaustive irradiation of FDA. In the next set of experiments, HepG2 cells treated with FDA were irradiated directly to find the immediate impact of the photochemical transformation of fluorescein to photoproducts, including ^1^O_2_ and CO. We observed a significant decrease in the intracellular concentrations of 2-hydroxyglutarate (2HG), 2-oxoglutarate (2OG), and citrate following FDA treatment in the dark. Incubation with fluorescein photoproducts negatively affected the cellular concentrations of 2HG and 2OG only. On the contrary, the irradiation of HepG2 cells treated with FDA resulted in a significant decrease in the majority of metabolites, with the most significant changes in the concentrations of lactate, 2HG, 2OG, and citrate (Figure 5A).

#### 2.4.2. CO Atmosphere

To test the hypothesis that the produced CO is responsible for decreased cell metabolism, HepG2 cells were incubated in an atmosphere containing 100 ppm of CO. A significant decrease in the concentrations of the Krebs cycle intermediates (2HG, glutamate, 2OG, and citrate) was observed following CO treatment for 2 h (Figure 5B).

### 2.5. Effect of Fluorescein Photochemical Reaction on Cell Cycle

**Figure 6:**
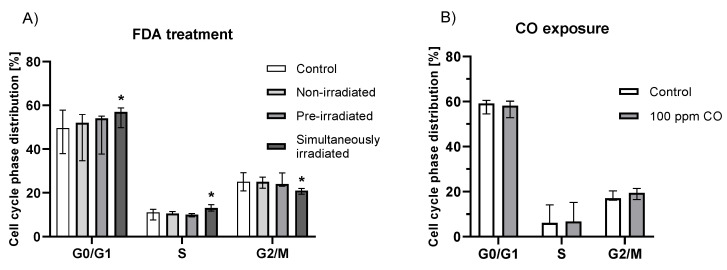
The effect of fluorescein irradiation (**A**) and elevated CO levels in the ambient atmosphere (**B**, 100 ppm) on the cell cycle of HepG2 cells. (**A**) Treated for 2 h with non-irradiated (c_FDA_ = 150 μmol/L), pre-irradiated (irradiation 24 h prior to treatment, c_FDA_ = 150 μmol/L, I = 160 mW/cm^2^) and simultaneously irradiated solutions of FDA (irradiation during the entire incubation time, I = 160 mW/cm^2^, c_FDA_ = 150 μmol/L); (**B**) cells exposed to an atmosphere enriched with gaseous CO (100 ppm) for 2 h.; (**A**) n = 9, (**B**) n = 12; * *p* ≤ 0.05 vs. controls (control = untreated cells).

Irradiation of HepG2 cells treated with FDA significantly affected the cell cycle progression toward slower cell division, as seen by an increase in the G0/G1 and S phases together with a decrease in the G2/M phase (Figure S4). No effect on the cell cycle was observed upon treatment with non-irradiated and pre-irradiated solutions of FDA or after cell incubation in a 100 ppm CO atmosphere (Figure 6).

## 3. Discussion

Fluorescein is a relatively nontoxic compound (LD_50_ = 6.7 g/kg for rats [39]), and it is widely used in medicine for diagnostic purposes, as it exhibits strong fluorescence in aqueous media [1,2,4,6]. This dye is also used as a fluorescent label in target tissues [1,5]. Our in vitro study on HepG2 cells showed minimal toxicity of fluorescein. Low toxicity was also observed for its stable photoproducts, created by exhaustive FDA irradiation. However, simultaneous irradiation of cells treated with fluorescein led to a significant and time-dependent decrease in cell viability, suggesting that one or more photoproducts formed during irradiation, which were not present in the solutions upon exhaustive irradiation, were responsible for the observed cytotoxicity. We conclude that these species must be either volatile or short-lived (although reactive).

We assumed that one of these toxic products is ^1^O_2_, as fluorescein is known to photosensitize oxygen to ^1^O_2_ with a quantum yield in the 0.03 [40] to 0.06 [13] range in aqueous media. Since ^1^O_2_ is a very reactive species that can induce apoptosis [24] and necrosis [25], it might be responsible for the observed decrease in cell viability.

The other identified photoproduct was CO. We observed an increased content of CO in irradiated cells treated with fluorescein (Figure 1). The determined yield of CO (~40%, Table S2) is surprisingly very high, especially when considering that CO is a biologically active molecule and the fact that fluorescein is a clinically commonly used dye. CO can cause respiration inhibition, alter the function of hemoproteins [34,41,42], or generate oxidative stress [36]. These are all effects that can influence cell viability. Therefore, CO formation might contribute significantly to the cytotoxicity of fluorescein during its irradiation.

Because fluorescein cannot cross the plasma membrane of cells very efficiently, we used its derivative, fluorescein diacetate (FDA), to investigate the effect of intracellular fluorescein localization. Comparing the effects of these two modes of fluorescein treatment helped us to assess the biological effects of ^1^O_2_ and CO when produced both intra- and extracellularly. Comparisons of the cell viability indicated that when administered as a free acid, fluorescein’s negative impact on viability is significantly smaller. In this case, the ^1^O_2_ molecules released during the photoreaction do not necessarily reach the intracellular compartment because of the short half-life of ^1^O_2_ (τ_1/2_ = 3–4 μs [43]). On the other hand, the long-lived CO (τ_1/2_ = 3–4 h) can freely pass through the plasma membrane and affect cellular processes when generated extracellularly, as shown by Lazarus et al. [44], who studied the intra- vs. extracellular delivery of CO using two types of CO-releasing molecules (CORMs) differing in their cellular localization. They showed that extracellular CO production exhibited a lower toxic effect on cells, whereas anti-inflammatory cell signaling processes were similar to those of intracellular delivery.

Experiments performed in a hypoxic chamber (a 9% O_2_ level was set according to the measured O_2_ level in rat livers [45]) showed that hypoxia was associated with a significantly lower drop in the viability of cells treated with either fluorescein or FDA when compared with cells under normoxic conditions. We propose three different ways that the O_2_ level may influence this parameter. A lower O_2_ level can result in: a lower yield of ^1^O_2_ (fewer O_2_ molecules available for sensitization) (Figure S3); reduced efficiency of the fluorescein photoreaction (if ^1^O_2_ is responsible for its degradation) and thus less efficient CO release; or a different cellular metabolic status, any of which ultimately affects the cell’s survival.

We also found that treatment of HepG2 cells with non-irradiated and pre-irradiated solutions only containing fluorescein or its photoproducts caused a decrease in Krebs cycle metabolite concentrations; a more profound decrease was observed upon irradiation of the cells treated with FDA (significantly lower concentrations of all Krebs cycle intermediates (*p* ≤ 0.05), with the exception of malate and fumarate, when compared to FDA and photoproducts). This indicates that, to some extent, fluorescein itself but primarily the above-mentioned biologically active by-products of fluorescein photoexcitation might affect the overall cellular energetic metabolism. To investigate the role of CO, HepG2 cells were exposed to an atmosphere containing 100 ppm CO, confirming the key role of this molecule in this process. These results correspond to those observed upon CO exposure that demonstrated the inhibition of respiration and glycolysis and a decrease in some Krebs cycle metabolites [46]. On the other hand, some published data have proved that CO can promote oxidative phosphorylation [47,48], mitochondrial biogenesis [49], and even an increase in cytochrome c oxidase activity [50], suggesting that the effect of CO is concentration- and tissue-dependent and reflects the overall cell/tissue status or oxygen level. In our case, significant inhibition of the Krebs cycle was observed, which coincided with a relatively high FDA concentration and a long exposure to generated and simultaneously irradiated fluorescein.

Irradiation of FDA-treated cells also resulted in a significant increase in the G0 phase and a simultaneous decrease in G2/M, indicating reduced proliferation and thus the antiproliferative and anticancer potential of fluorescein. To assess the function of CO, an analogous experiment was performed under a CO atmosphere. No significant effect of CO was observed on cell cycle progression, indicating no involvement of the CO released during the photoreaction in this process. However, CO has been suggested to affect the cell cycle [51,52], showing that this effect might be both dose- and cell-dependent.

In this paper, we show that irradiation of fluorescein solutions leads to the production of ^1^O_2_ and CO, which affects cell viability, metabolism, and proliferation. Incubation of cells with either fluorescein or FDA enabled us to observe the biological consequences of the extra- or intracellular release of fluorescein photoproducts, especially ^1^O_2_, whose short half-life limits its reactivity to the vicinity of its formation [53]. On the other hand, the long half-life of CO allows the molecule to freely pass through biological membranes, to reach dynamic equilibrium, and to affect cells regardless of its production site [44]. Nevertheless, there are some limitations in our study. As we do not know the exact mechanism of fluorescein photodegradation, other as yet unidentified photoproducts might contribute to the observed biological effects. In fact, we recently described the production of phthalic and formic acids upon fluorescein irradiation [37], which supports this assumption. Moreover, we could not clearly distinguish between the individual contributions of ^1^O_2_ and CO on cellular toxicity and metabolism, as their effects might overlap. It is important to point out that this study was conducted using the HepG2 cell line; however, the effect of fluorescein might be cell- and tissue-dependent. Further studies are needed to clarify these issues.

To conclude, we demonstrate that irradiation of fluorescein results in the production of the biologically active molecules ^1^O_2_ and CO. These molecules might be responsible for the phototoxicity of fluorescein, which increases with increasing dosage, times of irradiation, and tissue oxygenation levels. Moreover, the fluorescein photoreaction products affect cell metabolism and proliferation. As it releases CO in substantial amounts, it might even be used therapeutically as a photoCORM to release CO into target tissues irradiated with light.

## 4. Materials and Methods

### 4.1. Chemicals

All chemicals were obtained from Sigma-Aldrich (St. Louis, MO, USA), the organic solvents were from Penta (Prague, Czech Republic), and the cell culture reagents were from Biosera (Nuaillé, France) unless otherwise specified.

### 4.2. Cell Cultures

The human hepatoblastoma HepG2 cell line was purchased from the American Type Culture Collection (ATCC, Manassas, VA, USA) and cultured in supplemented Minimum Essential Medium (MEM) according to the manufacturer’s instructions in a humidified atmosphere containing 5% (*v*/*v*) CO_2_ at 37 °C. MEM medium without phenol red (Sigma-Aldrich, Saint-Louis, MO, USA) was used for all fluorescein solutions to prevent phenol red from affecting the irradiation experiments.

### 4.3. CO Content in HepG2 Cells

#### 4.3.1. Treatment and Sample Preparation

For analysis of the CO content, HepG2 cells were seeded on Petri dishes (d = 10 cm) and cultured according to the manufacturer´s protocol in supplemented MEM media at 37 °C and 5% (*v*/*v*) CO_2_; cells were then treated with FDA solution (c = 600 μmol/L, Hanks’ balanced salt solution (Hanks) with 5% (*v*/*v*) DMSO) for 30 min. Control cells were treated with Hanks containing only 5% (*v*/*v*) DMSO. The dishes were then washed with PBS, and 5 mL of Hanks was added. Dishes treated with FDA were irradiated for 2 h (LED, white light, I = 600 mW/cm^2^) or left in the dark for this period of time. The cells were then scraped from the surface of the dish, resuspended in 1 mL of PBS, centrifuged, and homogenized in 100 μL of PBS.

#### 4.3.2. GC-RGA Analysis

Cell homogenate was injected into septum-sealed and deaerated vials containing 10 μL of 60% 5-sulfosalicylic acid, and the CO released into the vial’s headspace was analyzed using gas chromatography with a reduction gas analyzer (GC-RGA Peak Performer 1, Peak Laboratories, Mountain View, CA, USA) as previously described [54].

### 4.4. Cell Viability

Cell viability was assessed by an MTT (3-(4,5-dimethylthiazol-2-yl)-2,5-diphenyltetrazolium bromide) reduction assay in 96-well plates. Cells were incubated in an MTT solution (50 μL, c = 6 g/L, MEM medium) for 15–30 min; 50 μL of DMSO was added to dissolve the formed formazan, and absorbance of the obtained solution was measured (λ_abs_ = 570 nm, Tecan, Infinite M200, Männedorf, Switzerland).

#### 4.4.1. Cytotoxicity of Fluorescein and Its Photoproducts

For the cytotoxicity analysis of fluorescein diacetate (FDA) [38,55] as well as its photoproducts, two incubation intervals (2 and 24 h) were used. A solution of the photoproducts (pre-irradiated solution) was prepared by exhaustive irradiation (t_ir_ = 24 h, prior to the treatment; I = 160 mW/cm^2^) of an FDA stock solution (c = 12 mM, MEM medium, dissolved in DMSO, 5%, *v*/*v*). Cells were treated with the studied solutions at concentrations of 75–2400 μmol/L diluted in a MEM medium for either 2 or 24 h, and the cell viability was analyzed. Control cells were treated with media without active substance and kept in the dark or irradiated for 2 or 24 h. No difference in cell viability was observed between the irradiated and non-irradiated controls.

#### 4.4.2. Cytotoxicity of Photoproducts

In the first set of experiments, the cells were treated with FDA (75–2400 μmol/L) and irradiated with white light (I = 160 mW/cm^2^) for 2 or 24 h. The MTT assay was performed immediately after irradiation.

The second set of experiments consisted of cells treated with FDA or fluorescein solutions within a concentration range of 9–300 μmol/L, irradiated for 30 min, and then analyzed immediately (or after additional incubation for 1.5 or 23.5 h in the dark). This experiment was also performed under hypoxic conditions (hypoxic chamber InvivO2^®^ 400 Physoxia Workstation, Baker Russkin, Sanford, ME, USA). The environmental parameters in the hypoxic chamber were maintained at: 9% O_2_, 5% CO_2_, t = 37 °C, and 70% relative humidity. Cells were seeded on a 96-well plate, incubated overnight in an incubator with normal O_2_ levels, and then put into the hypoxic chamber, where they were treated and incubated with the studied solutions after reaching 80% confluence. Cell viability was determined by the MTT assay performed under ambient atmosphere.

### 4.5. Determination of Intracellular Krebs Cycle Metabolites

#### 4.5.1. Cell Treatment and Sample Preparation

For analysis of the intracellular metabolites, cells were seeded on a Petri dish (d = 6 cm) and treated with the non-irradiated, pre-irradiated, and simultaneously irradiated solutions of FDA (c = 150 μmol/L, prepared as described earlier) for 2 h. Cells were trypsinized, washed with Hanks’ solution, and centrifuged (250 g, 5 min). The pellet was extracted with a water/methanol/chloroform mixture (1:1:2, *v*/*v*/*v*, 2 mL per sample) with the addition of sodium oxalate (c_final_ = 37 μmol/L) used as an internal standard and also EDTA (c_final_ = 25 μmol/L). The resulting suspension was centrifuged, and the aqueous phase was separated, lyophilized, and subsequently derivatized with a pyridine/*N*,*O*-bis(trimethylsilyl)acetamide/chlorotrimethylsilane mixture (10:4:2, *v*/*v*/*v*; 150 μL per sample) at 65 °C for 90 min.

#### 4.5.2. GC-MS Analysis

The prepared samples were analyzed using gas chromatography coupled with a mass spectrometer (GC-MS, GC 6890N, MD 5973, Agilent Technologies, Santa Clara, CA, USA) [56]. The following metabolites of the Krebs cycle and its anaplerotic pathways together with lactate were analyzed. The instrument was set to a SIM mode for the analysis, and the measured metabolites´ levels were normalized to that of the internal standard and the cell count (m/z of the fragments: oxalate—190; citrate—273; malate—355; 2HG and glutamate—349; 2OG—347; fumarate—245; lactate—219).

### 4.6. Cell Cycle Analysis

HepG2 cells were seeded on a Petri dish (d = 6 cm) and treated with the non-irradiated (normal FDA solution), pre-irradiated, and simultaneously irradiated solutions of FDA (c = 150 μmol/L) for 2 h, trypsinized, washed with ice-cold PBS, and centrifuged (250 g, 5 min). Cells in the pellet were fixed with 70% ethanol, incubated at 4 °C for at least 2 h, and treated with RNase (130 mg/L) for 15–30 min and propidium iodide (Thermo Fisher Scientific, Waltham, MA, USA) solution (100 μmol/L). Lastly, the cell suspension was centrifuged (850 g), resuspended in 1 mL of PBS, and analyzed using a flow cytometer (Mindray, BriCyte E6, Shenzhen, China) with MRFlow software (01.12.00.9280, Shenzhen, China). The wavelength used for the excitation was λ_ex_ = 488 nm, and the emission was measured with an FL2/PE channel (λ_em_ = 585 nm).

### 4.7. CO Exposure

To expose cells to the elevated atmospheric concentration of CO, we used an incubator equipped with an OxyCycler GT4181 (Biospherix, Parish, NY, USA). The CO level inside the chamber was set to 100 ppm. For the analysis of Krebs cycle metabolites and the cell cycle, the cells were incubated under these conditions for 2 h, which was followed by sample preparation and analysis, as described above.

### 4.8. Statistical Analyses

Normally distributed data are presented as a mean ± SD, while non-normally distributed data are shown as a median ± IQR. Comparisons were assessed by Student t-test or Mann–Whitney U test, respectively. To analyze differences within the groups, a conventional ANOVA test together with a Holm–Sidak post hoc multiple comparison test was used. The level of statistical significance was set to *p* ≤ 0.05.

## Data Availability

Not applicable.

## Supplementary Materials

The following supporting information can be downloaded at: https://www.mdpi.com/article/10.3390/ijms23031504/s1.
