# Supplementary material for: Antiproliferative and Cytotoxic Activities of Fluorescein—A Diagnostic Angiography Dye
Source: Int J Mol Sci. 2022 Jan 28;23(3):1504. doi: 10.3390/ijms23031504 (PMC8836159; doi:10.3390/ijms23031504)
Supplement: Supplementary file 1 [file ijms-23-01504-s001.zip › ijms-1558288-supplementary.pdf]

Article

Antiproliferative and Cytotoxic Activities of Fluorescein—A Diagnostic Angiography Dye

Mária Šranková [1], Aleš Dvořák [1], Marek Martínek [2], Peter Šebej [2], Petr Klán [2,3], Libor Vítek [1,4,*] and Lucie Muchová [1,*]

[1] Institute of Medical Biochemistry and Laboratory Diagnostics, General University Hospital in Prague and 1st Faculty of Medicine, Charles University, Na Bojišti 3, 121 08 Praha, Czech Republic; majka.srankova@gmail.com (M.Š.); aleshdvorak@gmail.com (A.D.)

[2] RECETOX, Faculty of Science, Masaryk University, Kotlářská 2, 611 37 Brno, Czech Republic;
marek.martinek@gmail.com (M.M.); sebej@recetox.muni.cz (P.Š.); klan@sci.muni.cz (P.K.)

[3] Department of Chemistry, Faculty of Science, Masaryk University, Kamenice 5, 625 00 Brno, Czech
Republic;

[4] 4th Department of Internal Medicine, General University Hospital in Prague and 1st Faculty of Medicine, Charles University, U Nemocnice 2, 128 08 Praha, Czech Republic

* Correspondence: lucie.muchova@lf1.cuni.cz (L.M.); vitek@cesnet.cz (L.V.); Tel.: +42-022-496-4199 (L.M.)

Materials and Methods

Singlet oxygen production

Normoxia

To study the production of $^1O_2$, we used 9,10-anthracenediyl-bis(methylene)dimalonic acid (ABDA) as a spectrophotometric probe. For the evaluation of $^1O_2$ quantum yield, the decrease in the absorbance of ABDA at its absorption maximum (λ_{abs}(max) = 381 nm) upon irradiation of the compound of interest with white light (4 intervals, t_{int} = 7 min, I = 350 mW/cm^2) was analyzed. Rose Bengal (RB) was used as a standard [1,2]. Stock solutions of fluorescein disodium salt (c = 7.5 mmol/l, DMSO, ABDA (c = 2 mmol/l, DMSO) and RB (c = 750 µmol/l, PBS) were stored in a freezer (−18 °C) for a short-term basis. The final concentration of photosensitizers in the tested sample was 1.4 µmol/l (PBS, 7% (v/v) DMSO) and 140 µmol/l (PBS, 7% (v/v) DMSO) for ABDA. Experiments were performed in quadruplicates.

The quantum yield of $^1O_2$ generated upon sensitization was calculated using Eq. (1) [3]:

$$\varphi_A = \varphi_{RB} \times \frac{S_A\, I_{RB}^{ABS}}{S_{RB}\, I_A^{ABS}} \ ,$$

where φ is the $^1O_2$ quantum yield of the photosensitizer, S is the rate of consumption (decay) of the $^1O_2$ acceptor in the presence of the tested compound, and I^{ABS} is the amount of light quanta absorbed by the photosensitizer (either standard, RB, or tested compound). I^{ABS} was calculated as an integral of the product of relative light source intensity, and the ratio of absorbed and incident radiant flux of the tested sample with respect to the wavelength. S_A and S_{RB} were calculated with using least square regression.

Hypoxia

The $^1O_2$ experiment was also performed in a hypoxic chamber (InvivO2® 400 Physoxia Workstation, Baker Ruskinn, Sanford, ME, USA). After analogous solution preparation under normal atmosphere, the irradiation and measurements were carried out in the hypoxic chamber with the following parameters: 9% (*v*/*v*) O_2, 1.5% (*v*/*v*) CO_2, t = 32 °C and relative humidity RH = 50%. Only fluorescein solutions were measured in this experimental design, without the use of RB as a standard. Relative values of ABDA´s decrease in absorbance were compared with results obtained under normal atmosphere to evaluate the impact of O_2 level on $^1O_2$ production.

Quantification of released CO

GC-MS analysis

Aerated reaction mixtures of fluorescein (c ~ 2 × 10^{-5} mol/l , V = 0.5 ml) in aqueous solution (PBS buffer, pH = 7.4, I = 0.1 mol/l) in a 2 mL head-space vial with a gas-tight septum were irradiated using three 100 W white LED lamps for 4 days (full conversion; judged by the disappearance of the starting material, as determined by disappearance of the typical spectral features in the visible light portion of the respective absorption spectra), and an aliquot (100 μl) of the gaseous phase over the reaction mixture was analyzed by gas chromatography coupled with a mass spectrometry detector (GC with MS). The values obtained were at least four orders of magnitude higher than background CO (g) levels (in the ambient air), and thus the obtained values were not corrected to the background.

GC-RGA analysis

A solution of fluorescein in PBS buffer (c = 150 μmol/l, V = 100 μl) was exhaustively irradiated by white light (t = 16 h) in clear CO-free septum-sealed vials. CO released into the vial headspace was measured using gas chromatography with a reduction gas analyzer (GC-RGA Peak Performer 1, Peak Laboratories, CA, USA) as previously described [4].

The measured values were compared with the theoretical yields using the assumption of one CO molecule released from each degraded molecule of fluorescein dye molecule.

Fluorescence microscopy

To visualize fluorescein cellular localization, human hepatoblastoma HepG2 cells were seeded into a 24-well plate and cultured according to the manufacturer´s protocol. At time t = 0, growth media were replaced for fluorescein (c = 150 μmol/l) or FDA (c = 150 μmol/l, MEM with 1% (*v*/*v*) DMSO as a co-solvent) and incubated for 30 min at 37 °C and 5% (*v*/*v*) CO_2. Then, the cells were washed 3× with PBS, and visualized using fluorescence microscopy (Olympus IX 51 with mercury burner U-RFL-T, Olympus, Japan). Selected areas from each well were photographed under 20× magnification and fixed exposure times using a WIB 520 nm emission filter.

A)

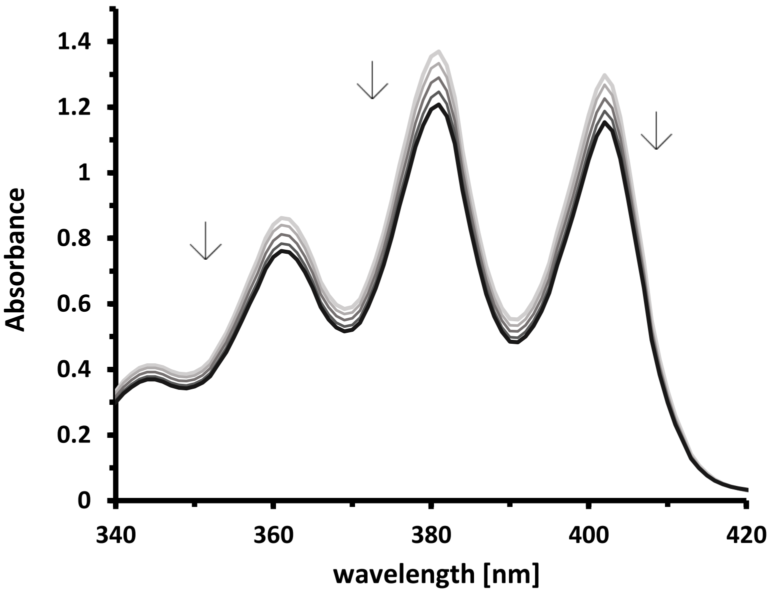

B)

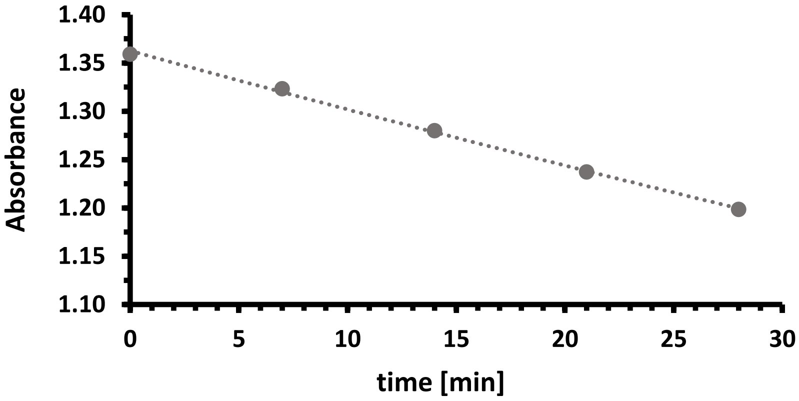

Figure S1. Determination of $^1O_2$ production upon irradiation of fluorescein (21% O_2, PBS, c(ABDA) = 140 μmol/l, c(FL) = 1.4 μmol/l, I = 350 mW/cm^2). **A**) Change in absorption spectra of ABDA and fluorescein solution over time upon irradiation with white light; **B**) Absorption change at λ_{abs} = 381 nm (corresponding to the absorption maximum of ABDA) over time upon irradiation together with a linear function fit; ABDA - 9,10-anthracenediyl-bis(methylene)dimalonic acid; arrows indicate the direction of change of the absorption spectra. Spectra were recorded every 7 min.

Table S1. **$^1O_2$ quantum yield of fluorescein** (21% (v/v) O_2, PBS, c(FL) = 1.4 μmol/l, I = 350 mW/cm^2) determined with the use of an ABDA spectrophotometric probe (c(ABDA) = 140 μmol/l) and a standard, RB ($\phi(^1O_2)$ = 0.75[1]) compared with values from the literature.

Fluorescein	Our result	Literature	
$^1O_2$ quantum yield $\phi(^1O_2)$	0.015-0.028	0.03[1]	0.06[5]

Table S2. Yield of CO released upon irradiation of fluorescein (c = 150 μmol/l) in PBS (100 μl) measured by means of gas chromatography coupled with a reduction gas analyzer (GC-RGA) or mass spectrophotometry (GC-MS).

Type of analysis	GC/RGA	GC/MS
CO yield (%)	41±11	39±13

5ms **10ms** **50ms** **100ms** **200ms**

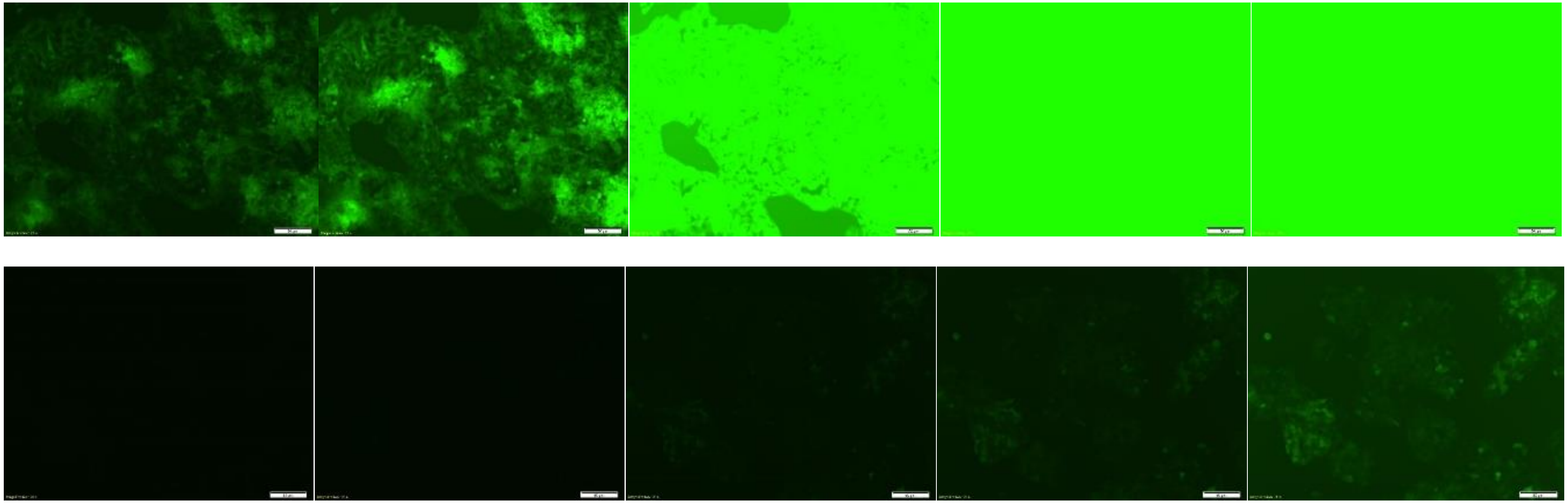

Figure S2. Fluorescent microscopy images of HepG2 cells treated with FDA (c = 150 µmol/l, top) or fluorescein (c = 150 µmol/l, bottom) for 30 minutes. Fixed exposure times were set for 5, 10, 50, 100 or 200 ms to compare the fluorescence of the cells. The scale bar represents 50 µm.

International Journal of
Molecular Sciences

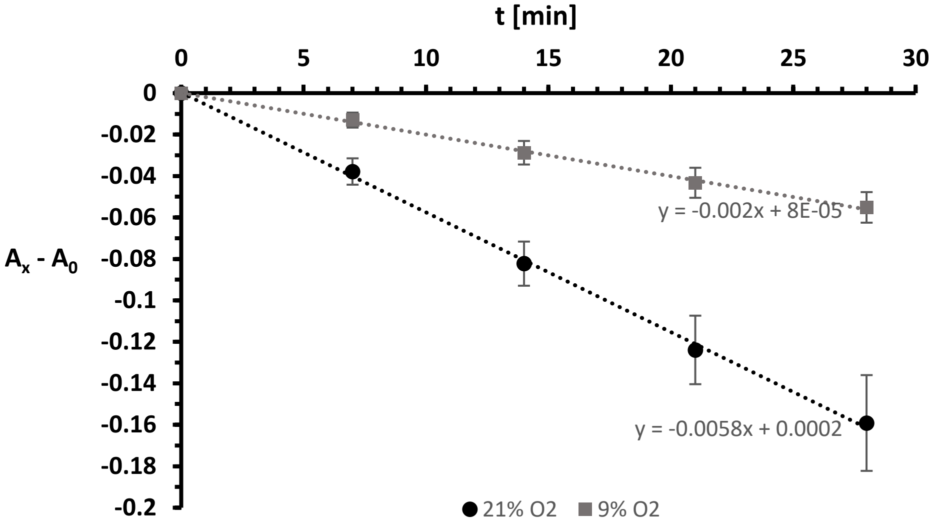

Figure S3. Absorption change of ABDA and fluorescein solution upon irradiation under normoxic and hypoxic conditions. Absorption change at λ_{abs} = 381 nm (ABDA absorption maximum) of ABDA and fluorescein solution upon irradiation under normoxic (black circle) and hypoxic (grey square) conditions (PBS, c(ABDA) = 140 µmol/l, c(FL) = 1.4 µmol/l, I = 350 mW cm^{-2} white light). Spectra were recorded every 7 min; n = 8; A_x, absorbance at t = x; A_0, absorbance at t = 0.

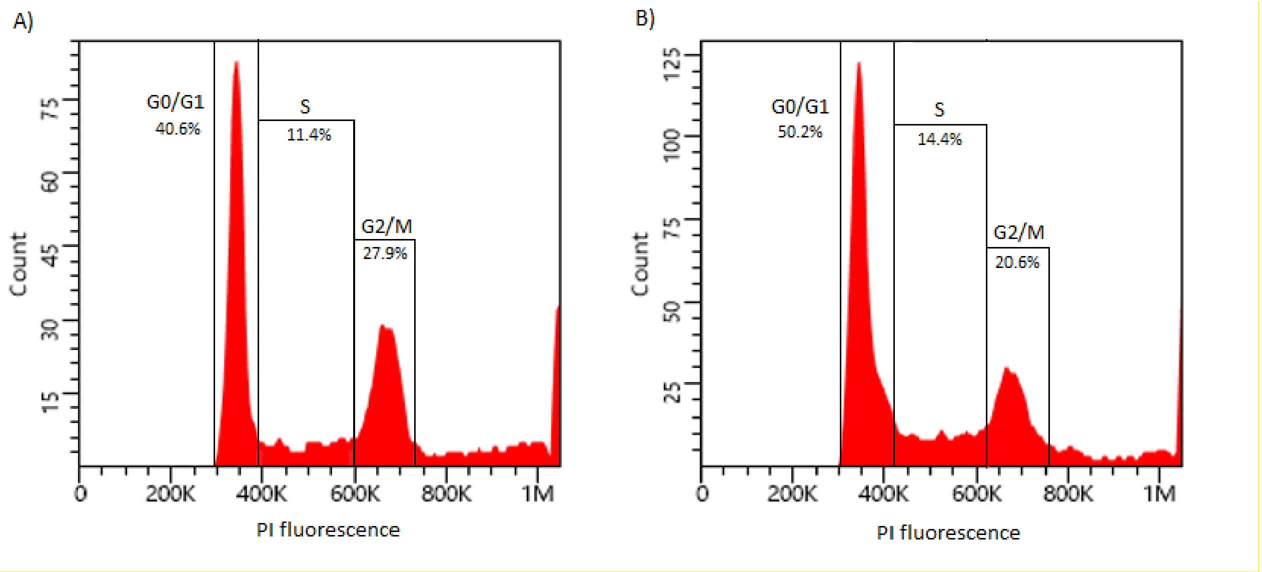

Figure S4. Histograms of the cell cycle distribution of control cells (A) and cells treated with FDA and simultaneously irradiated for 2 h (I = 160 mW/cm^2, c_{FDA} = 150 µmol/l. Graphs represent the distribution of cells ("Count") according to the fluorescence intensity of used DNA stain ("PI fluorescence") corresponding to their DNA content; PI, propidium iodide.

References

1. Gandin, E.; Lion, Y.; Van de Vorst, A. Quantum yield of singlet oxygen production by xanthene derivatives. *Photochemistry and photobiology* **1983**, *37*, 271–278.
2. Lutkus, L.V.; Rickenbach, S.S.; McCormick, T.M. Singlet oxygen quantum yields determined by oxygen consumption. *Journal of Photochemistry and Photobiology A: Chemistry* **2019**, *378*, 131–135.
3. Kuznetsova, N.; Gretsova, N.; Yuzhakova, O.; Negrimovskii, V.; Kaliya, O.; Luk'Yanets, E. New reagents for determination of the quantum efficiency of singlet oxygen generation in aqueous media. *Russian Journal of General Chemistry* **2001**, *71*, 36–41.
4. Vreman, H.J.; Stevenson, D.K. Heme oxygenase activity as measured by carbon monoxide production. *Analytical biochemistry* **1988**, *168*, 31–38.
5. Usui, Y. Determination of quantum yield of singlet oxygen formation by photosensitization. *Chemistry Letters* **1973**, *2*, 743–744.
